# M1 Muscarinic Receptor Deficiency Attenuates Azoxymethane-Induced Chronic Liver Injury in Mice

**DOI:** 10.1038/srep14110

**Published:** 2015-09-16

**Authors:** Vikrant Rachakonda, Ravirajsinh N. Jadeja, Nathalie H. Urrunaga, Nirish Shah, Daniel Ahmad, Kunrong Cheng, William S. Twaddell, Jean-Pierre Raufman, Sandeep Khurana

**Affiliations:** 1 Division of Gastroenterology and Hepatology, University of Maryland School of Medicine, Baltimore, Maryland, 21201; 2 Section of Gastroenterology and Hepatology, Georgia Regents University, Augusta, GA 30912; 3 Department of Pathology, University of Maryland School of Medicine, Baltimore, Maryland, 21201

## Abstract

Cholinergic nervous system regulates liver injury. However, the role of M1 muscarinic receptors (M1R) in modulating *chronic* liver injury is uncertain. To address this gap in knowledge we treated M1R-deficient and WT mice with azoxymethane (AOM) for six weeks and assessed liver injury responses 14 weeks after the last dose of AOM. Compared to AOM-treated WT mice, M1R-deficient mice had attenuated liver nodularity, fibrosis and ductular proliferation, α-SMA staining, and expression of *α1 collagen*, *Tgfβ-R, Pdgf-R, Mmp-2, Timp-1* and *Timp-2.* In hepatocytes, these findings were associated with reductions of cleaved caspase-3 staining and *Tnf-α* expression. In response to AOM treatment, M1R-deficient mice mounted a vigorous anti-oxidant response by upregulating Gclc and Nqo1 expression, and attenuating peroxynitrite generation. M1R-deficient mouse livers had increased expression of *Trail-R2*, a promotor of stellate cell apoptosis; dual staining for TUNNEL and α-SMA revealed increased stellate cells apoptosis in livers from M1R-deficient mice compared to those from WT. Finally, pharmacological inhibition of M1R reduced H_2_O_2_-induced hepatocyte apoptosis *in vitro*. These results indicate that following liver injury, anti-oxidant response in M1R-deficient mice attenuates hepatocyte apoptosis and reduces stellate cell activation, thereby diminishing fibrosis. Therefore, targeting M1R expression and activation in chronic liver injury may provide therapeutic benefit.

Chronic liver injury is characterized by hepatic fibrosis, resulting from a detrimental wound healing response involving replacement of laminar basement membrane Type IV collagen with a fibrillar scar matrix composed primarily of Type I and Type III collagen^1^. If unabated, fibrosis progresses to cirrhosis, the common final pathway for chronic liver disease and liver cancer. Current remedies for hepatic fibrosis are limited to treating the underlying disease, e.g. infection with hepatitis C, or if that fails, liver transplantation. As the incidence of chronic liver disease and cirrhosis has risen, the need for effective anti-fibrotic therapy has become crucial.

The autonomic nervous system may provide a therapeutic target for chronic liver injury. Sympathetic and cholinergic innervation to the liver undergo substantial modifications in hepatic fibrosis; for example, cholinergic fiber density increases in fibrotic liver^2^. In mice, both chemical sympathectomy and inhibiting α_1_ receptor adrenergic signaling reduce hepatic fibrosis following carbon tetrachloride (CCl_4_)-induced liver injury^3^. In contrast, treatment with norepinephrine increases hepatic fibrosis and collagen deposition in a murine model of diet-induced steatohepatitis^4^. Finally, in *mdr2−/−* mice that are prone to developing sclerosing cholangitis, nonspecific inhibition of β-adrenergic receptor signaling with propranolol ameliorates hepatic fibrosis^5^.

Recent evidence suggests that the parasympathetic/cholinergic nervous system also modulates liver injury. The liver receives cholinergic input from hepatic branches of the vagus nerve; acetylcholine is the primary neurotransmitter responsible for signal transmission. Acetylcholine can bind two types of cholinergic receptors: nicotinic receptors that function as ion channels, and muscarinic receptors that function by coupling to G-proteins. Five muscarinic receptors subtypes, designated M1R-M5R, participate in both neurotransmission and mediation of non-neuronal end-organ function^6^.

Disrupted vagal innervation in the transplanted liver may alter pre- and post-transplant liver disease phenotypes. When compared to innervated controls, denervated human livers affected with chronic hepatitis had diminished reactive bile duct proliferation and hepatic progenitor cell expansion^7^. In rats, vagus nerve transection decreased oval cell number after galactosamine-induced hepatic injury^7^. In vagotomized mice, bile duct ligation (BDL) increased cholangiocyte apoptosis and decreased cholangiocyte proliferation^8^. In a murine model of CCl_4_-induced chronic liver injury, both pharmacological inhibition of vagal signaling with atropine and surgical vagotomy reduced hepatic fibrosis compared to controls^9^. While these and other studies provide clear evidence of vagal modulation of chronic liver injury, because the vagus nerve contains and releases multiple neurotransmitters, including acetylcholine, vasoactive intestinal peptide, and nitric oxide, the precise cellular mechanisms that mediate its effects are uncertain. Moreover, acetylcholine is a ligand for both muscarinic and nicotinic receptors.

M3 muscarinic receptors (encoded by *CHRM3*), expressed by both human and rodent liver, have emerged as potential regulators of chronic liver injury^7^. Using a murine model of azoxymethane (AOM)-induced chronic liver injury, we showed that genetic ablation of *Chrm3* exacerbates AOM-induced gross liver injury, hepatic fibrosis, ductular proliferation, and oval cell expansion^10^. Compared to control, hepatocytes from AOM-treated M3R-deficient mice evidenced increased apoptosis and reduced proliferation. While treatment of WT mice with scopolamine butylbromide, a nonselective muscarinic receptor antagonist, augmented AOM-induced chronic liver injury, the extent of injury was less than that observed in AOM-treated M3R-deficient mice^10^. These findings suggested to us that other muscarinic receptors, possibly M1R, might oppose M3R in modulating chronic liver injury.

M1R (encoded by *CHRM1*) is expressed widely in the gastrointestinal system, and it participates in similar downstream signaling pathways as M3R. M1R and M3R are commonly co-expressed in neural and non-neural tissues^11^. In neuronal and gonadal cell lines, M1R is implicated in regulating cell injury and death^12^,^13^. *CHRM1* mRNA has been isolated from whole human and rodent livers^7^. Recently, we demonstrated that M1R modulates acute acetaminophen-induced liver injury^14^. Based on these collective observations, we hypothesized that M1R play an important role in the development of chronic liver injury. To test this hypothesis, we compared AOM-induced chronic liver injury in M1R-deficient and control mice.

## Results

### After AOM administration, hepatic gross nodularity and fibrosis are significantly decreased in M1R-deficient mice

**Figure 1:**
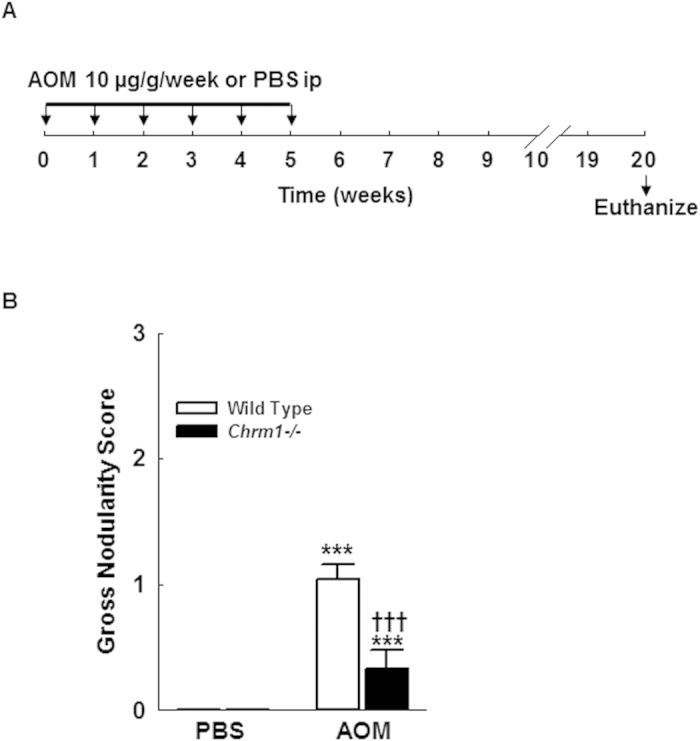
(**A**) Experimental design: 24 WT and 20 M1R-deficient mice were treated with 10 μg/g AOM i.p. once weekly for six weeks; 5 WT and 5 M1R-deficient mice were treated with PBS i.p. once weekly. Animals were sacrificed 20 weeks after the first AOM or PBS injection. (**B**) After AOM exposure, gross nodularity was significantly increased in WT but not M1R-deficient mice (Grade 0 = none, 1 = mild, 2 = moderate, 3 = marked and/or ascites). ***p < 0.001 within genotype and **†††***p* < 0.001 within treatment group.

**Figure 2:**
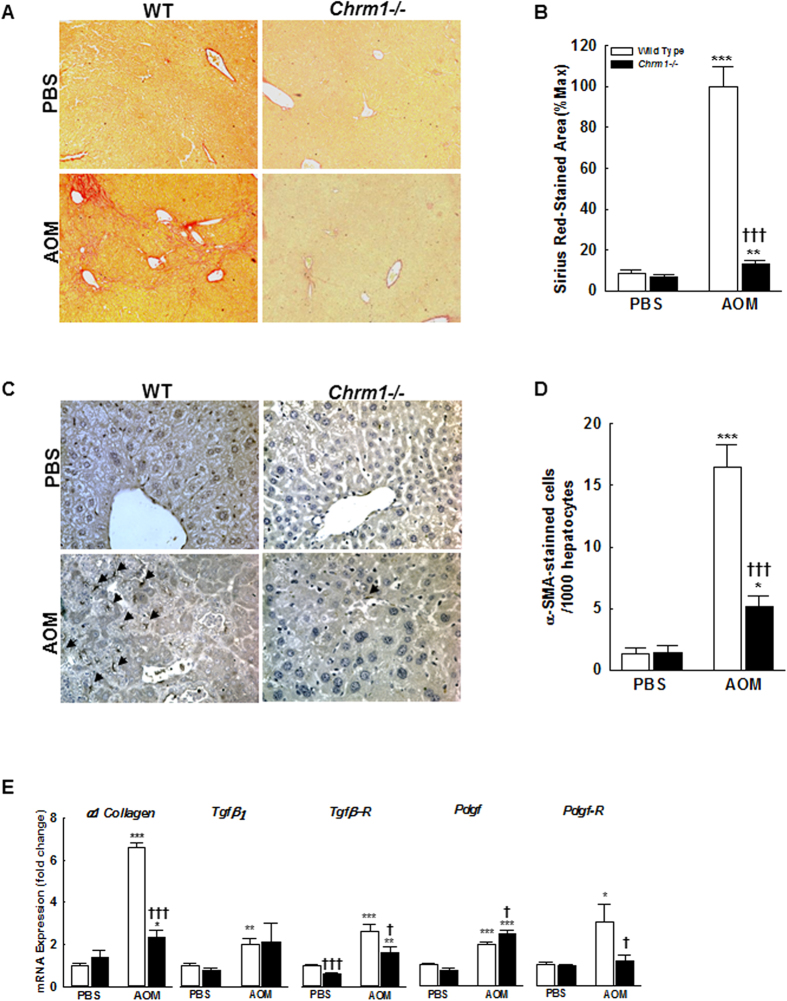
M1R deficiency inhibits AOM-induced hepatic fibrosis and HSC activation. (**A**,**B**) Sirius Red-stained liver sections from WT and M1R-deficient mice at 100x magnification. AOM increased liver fibrosis in both WT and M1R-deficient mice. Compared to AOM-treated WT mice, M1R-deficient mice had reduced Sirius Red staining (arbitrary units% max.). (**C**,**D**) α-SMA-stained liver sections from WT and M1R-deficient mice at 200x magnification. Arrows indicate α-SMA (+) HSC. Compared to AOM-treated WT mice, M1R-deficient mice had reduced α-SMA staining. M1R deficiency alters mRNA expression of HSC activation factors and their receptors, resulting in reduced *α1 Collagen* expression. Compared to AOM-treated WT mice, M1R-deficient mice had (**E**) decreased *α1 Collagen* mRNA expression, similar expression of *Tgf-β*_*1*_ and slightly increased expression of *Pdgf*, and decreased expression of *Tgfβ-R* and *Pdgf-R*. These findings suggest that M1R deficiency decreases HSC sensitivity to TGF-β and PDGF signaling. *p < 0.05, **p < 0.01, ***p < 0.001 within genotype, and **†***p* < 0.05, **†††***p* < 0.001 within treatment group.

Liver injury was not observed in PBS-treated M1R-deficient or WT mice and there were no differences in fibrosis as assessed by Sirius Red staining. In contrast, after repeated exposure to AOM, gross nodularity increased in WT, but not M1R-deficient mice (Fig. 1B). Compared to AOM-treated WT mice, M1R-deficient mice had ~87% reduced fibrosis (Fig. 2A,B); gross nodularity scores correlated positively with fibrosis (Pearson *r*^*2*^ = 0.82; *p* < *0.01*). As there were no baseline differences in Sirius Red staining or gross nodularity, increased liver injury seen in AOM-treated WT mice most likely indicates increased net collagen deposition.

Hepatic stellate cells (HSC) play a central role in the development of fibrosis. During chronic liver injury, HSC are activated from a quiescent state to a myofibroblastic phenotype. Perpetuation of HSC activation and proliferation are critical to maintenance of fibrosis, as activated HSCs are the primary source of collagen comprising the scar matrix. To determine the effect of M1R deficiency on HSC activation in AOM-induced chronic liver injury, we used IHC to measure expression of α-SMA, a marker of stellate cell activation. In PBS-treated mice, the baseline number of activated HSC in WT and M1R-deficient mice was similar. Following AOM exposure, activated HSCs were lower in M1R-deficient compared to WT mice (Fig. 2C,D). Further, α1 collagen(I) mRNA expression was increased to a greater degree in AOM-treated WT mice than in M1R-deficient mice (6.62 ± 0.19 vs. 2.33 ± 0.034 fold respectively, *p* < *0.001*; Fig. 2E).

We showed previously that in livers from WT and M1R-deficient mice there was no difference in mRNA expression of cytochrome P450 isozymes such as Cyp2e1 that participate in AOM metabolism^14^. This provides reassurance that these differences in liver injury do not result from altered AOM metabolism.

Transforming growth factor β (TGFβ) is an important cytokine secreted by HSC, injured hepatocytes, Kupffer cells, and other inflammatory cells in response to liver injury. TGFβ_1_ is the major isoform responsible for HSC activation and type I collagen production^15^. To assess the role of M1R on TGFβ-mediated HSC activation, we measured mRNA expression levels of TGFβ_1_ and its receptor (TGFβ-R). There was no difference in *Tgf-β*_*1*_ expression in WT and M1R-deficient mice following AOM or PBS treatment (Fig. 2E). However, compared to WT mice, *Tgfβ-R* expression was significantly reduced in M1R-deficient mice exposed to both PBS (1.0 ± 0.04 vs. 0.62 ± 0.03 fold respectively, *p* < *0.001*) and AOM (2.6 ± 0.3 vs. 1.6 ± 0.3 fold respectively, *p* < *0.05*; Fig. 2E), suggesting *Chrm1* gene ablation reduces HSC activation by attenuating TGFβ-R expression.

PDGF is the most potent HSC mitogen, signaling through both autocrine and paracrine pathways to promote activated HSC proliferation^16^. Increased PDGF receptor (PDGF-R) expression enhances HSC sensitivity to PDGF. We measured expression levels of mRNA for PDGF and PDGF-R. Compared to AOM-treated WT mice, M1R-deficient mice had slightly increased expression of *Pdgf* (2.0 ± 0.11 vs. 2.46 ± 0.16 fold respectively, *p* < *0.05*) but significantly decreased expression of *Pdgf-R* (3.05 ± 0.84 vs. 1.17 ± 0.28 fold respectively, *p* < *0.05*; Fig. 2E), suggesting that *Chrm1* deficiency reduces activated HSC proliferation in part through down-regulation of PDGF-R.

### Decreased fibrosis in M1R-deficient mice is mediated by altered expression of extracellular matrix (ECM) regulators

**Figure 3:**
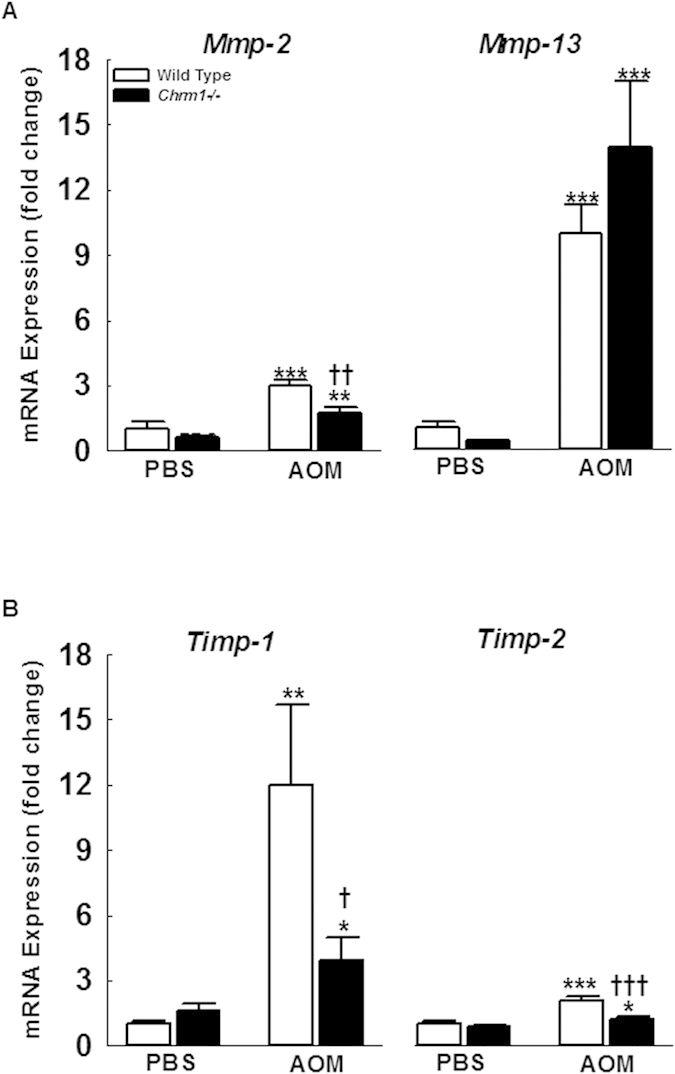
M1R deficiency alters AOM-induced mRNA expression of ECM modulators. (**A**,**B**) Compared to AOM-treated WT mice M1R-deficient mice had decreased expression of *Mmp-2*, *Timp-1* and *Timp-2.* M1R deficiency did not alter *MMP-13* expression. *p < 0.05, **p < 0.01, ***p < 0.001 within genotype, and **†***p* < 0.05, **††***p* < 0.01, **†††***p* < 0.001 within treatment group.

Activated HSC secrete matrix metalloproteinases (MMPs) and their tissue inhibitors (TIMPs) to promote replacement of laminar basement membrane collagen with fibrillar collagen. The interstitial collagenases MMP-2 and MMP-13 are upregulated during resolution of fibrosis, while TIMP-1 and TIMP-2 inhibit MMP activity. In murine models of fibrosis resolution, the relative balance of MMP and TIMP expression is shifted to promote collagen degradation^17^,^18^. Furthermore, murine studies of CCl_4_-induced liver injury demonstrated that TIMP-1 overexpression enhances fibrosis and inhibits fibrosis resolution^19^,^20^. TIMP-1 may also exert anti-apoptotic effects on activated HSC^20^,^21^. To assess the role of M1R in ECM remodeling, we measured expression levels of MMPs and their inhibitors (Fig. 3). In PBS-treated mice, there were no baseline differences in the expression of *Mmp-2, Mmp-13, Timp-1*, and *Timp-2*. Compared to AOM-treated WT mice, M1R-deficient mice had reduced expression of *Mmp-2* (2.95 ± 0.29 vs. 1.72 ± 0.26 fold respectively, *p* < *0.01*; Fig. 3A), *Timp-*1 (11.98 ± 3.67 vs. 3.92 ± 1.07 fold respectively, *p* < *0.05*), and *Timp-2* (2.08 ± 0.18 vs. 1.23 ± 0.13 fold respectively, *p* < *0.001*; Fig. 3B). M1R ablation did not alter *Mmp-13* expression. These findings are consistent with the increased HSC activation seen in WT mice, as activated HSC secrete MMPs and TIMPs to promote hepatic fibrosis.

### M1R deficiency reduces AOM-induced hepatocyte loss and bile ductular hyperplasia

Hepatic fibrosis develops in response to liver injury, and studies have demonstrated that fibrogenic mediators, such as Kupffer cells and hepatic stellate cells, undergo activation after phagocytosis of hepatocyte apoptotic bodies^22^,^23^. Furthermore, in human and experimental models of chronic liver injury, hepatocyte apoptosis and caspase activity correlate with histological severity of disease^24^,^25^. Fas ligand (FasL) and TNFα are key ligands that bind their respective cognate death receptors, Fas and TNFα-R1, to initiate apoptosis programs in hepatocytes^26^.

**Figure 4:**
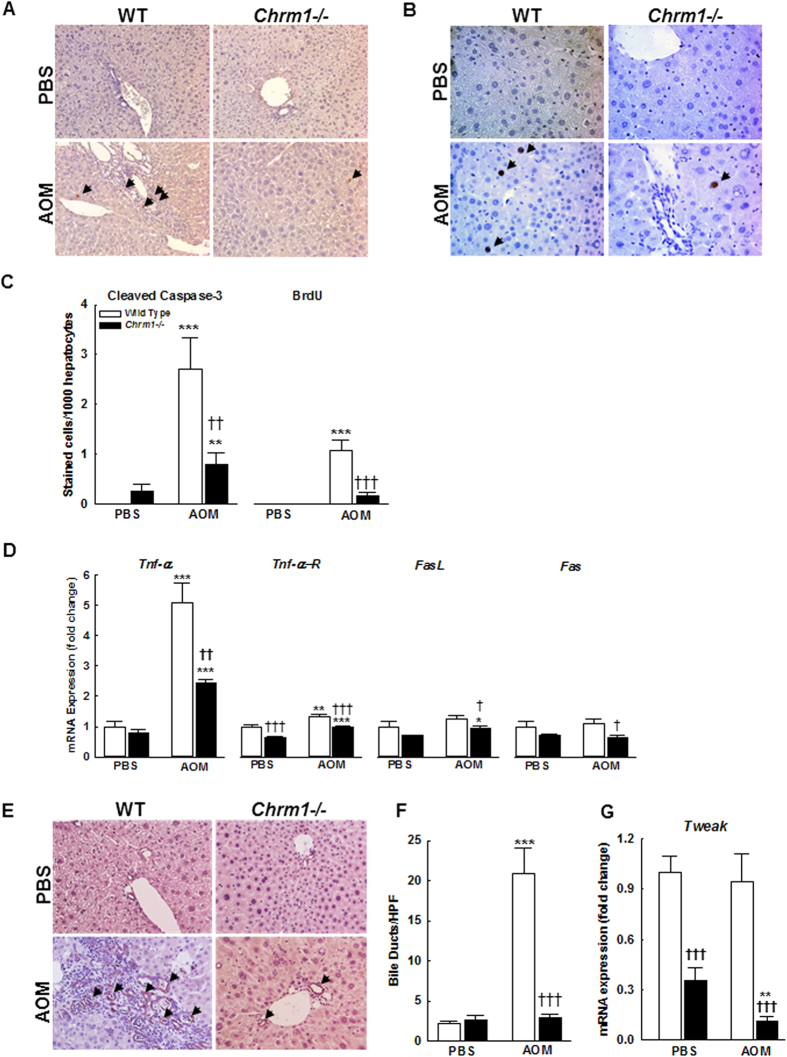
M1R deficiency reduces AOM-induced hepatocellular apoptosis and inhibits bile ductular hyperplasia. (**A**) Cleaved caspase-3-stained liver sections from WT and M1R-deficient mice at 200x magnification. Arrows indicate cleaved caspase-3-stained hepatocyte. (**B**) BrdU-stained liver sections from WT and M1R-deficient mice at 200x magnification. Arrows indicate BrdU-stained hepatocyte nuclei. (**C**) Compared to AOM-treated WT mice, M1R-deficient mice had reduced hepatocyte apoptosis and proliferation. M1R deficiency alters mRNA expression of hepatocellular death receptors and ligands. (**D**) Compared to AOM-treated WT mice, M1R-deficient mice had reduced *Tnf-α* expression. AOM increased *Tnf-α* expression in both groups. The differences in expression of *Tnf-αR1*, *FasL*, or *Fas* were modest. (**E**) CK-19-stained liver sections from WT and M1R-deficient mice at 200x magnification. AOM treatment-induced bile duct proliferation. (**F**) Compared to AOM-treated WT mice, M1R-deficient mice had markedly reduced bile-duct proliferation. (**G**) Compared to PBS-treated WT mice, M1R-deficient mice had reduced *Tweak* expression, which, upon AOM-treatment, decreased further. *p < 0.05, **p < 0.01, ***p < 0.001 within genotype, and **†***p* < 0.05, **††***p* < 0.01, **†††***p* < 0.001 within treatment group.

Hepatic repair after chronic liver injury is partially mediated by the balance between hepatocyte proliferation and apoptosis. To examine the role of M1R in hepatocyte apoptosis, we measured cleaved caspase-3 expression by immunohistochemistry. At baseline, there was no difference in cleaved caspase-3 expression. After AOM exposure, apoptosis was increased in both WT and M1R-deficient mice (Fig. 4A,C). To determine the role of M1R in hepatocyte proliferation we assayed BrdU staining by IHC. In PBS-treated mice, there was no baseline hepatocyte proliferation. With AOM treatment, hepatocellular proliferation was elevated in WT compared to M1R-deficient mice (Fig. 4B,C). Overall, M1R deficiency decreased hepatocyte apoptosis and reduced hepatocyte proliferation.

To assess the role of M1R in death receptor signaling, we measured expression of *Tnf-α, Tnf-αR1, Fas*, and *FasL* (Fig. 4D). Although *Tnf-α* was similarly expressed in PBS-treated mice, M1R deficiency reduced AOM-induced upregulation of TNFα (5.1 ± 0.64 vs. 2.42 ± 0.12 fold respectively, *p* < *0.01*; Fig. 4D). In PBS-treated mice, there were no baseline differences in *Fas* and *FasL* expression, and after AOM treatment, *Fas* and *FasL* expression were only mildly higher in WT mouse livers (Fig. 4D).

Bile duct hyperplasia is a well-described feature of AOM-induced chronic liver injury^10^,^27^,^28^. Ductular hyperplasia may promote hepatic fibrosis; several rodent studies showed that proliferating cholangiocytes secrete profibrogenic factors, including TGF-β and PDGF^29^,^30^. Furthermore, bile duct hyperplasia correlates with advanced fibrosis in human liver disease^31^,^32^,^33^. Though mechanisms underlying ductular reaction are poorly understood, recent reports suggest that TWEAK (TNF-related weak inducer of apoptosis) signaling plays a major role^34^,^35^. However, in a choline-deficient, ethionine-supplemented diet-induced liver injury and ductular reaction mouse model, there was no increase in hepatic *Tweak* expression^36^. Also, within 12 h after partial hepatectomy in mice, *Tweak* expression was markedly suppressed and recovered with liver regeneration^34^. Interestingly, in fibrotic mice that underwent 70% hepatectomy, inhibition of TWEAK signaling reduced the fibrogenic response and promoted liver regeneration^34^,^35^. Keeping these observations in mind, to assess the role of M1R on ductular hyperplasia, bile ducts were counted in liver sections; we confirmed the identity of bile ducts by staining for CK-19. Bile duct hyperplasia in AOM-treated WT mice was reduced with M1R deficiency (Fig. 4E,F); bile duct hyperplasia correlated positively with hepatic fibrosis (Pearson *r*^*2*^ = 0.734; *p* < *0.001*). Consistent with previous reports, there was no change in *Tweak* expression among PBS- and AOM-treated WT mice (Fig. 4G). However, *Tweak* expression was markedly reduced in PBS-treated M1R-deficient mice (1.0 ± 0.09 vs. 0.36 ± 0.07 fold respectively, *p* < *0.001*); this decreased further following treatment with AOM (0.11 ± 0.03 fold, *p* < *0.01*). These findings show that M1R deficiency prevents AOM-induced ductular hyperplasia, thereby likely preventing fibrosis.

### M1R deficiency reduces AOM-induced oxidative stress by promoting anti-oxidant response in mice

**Figure 5:**
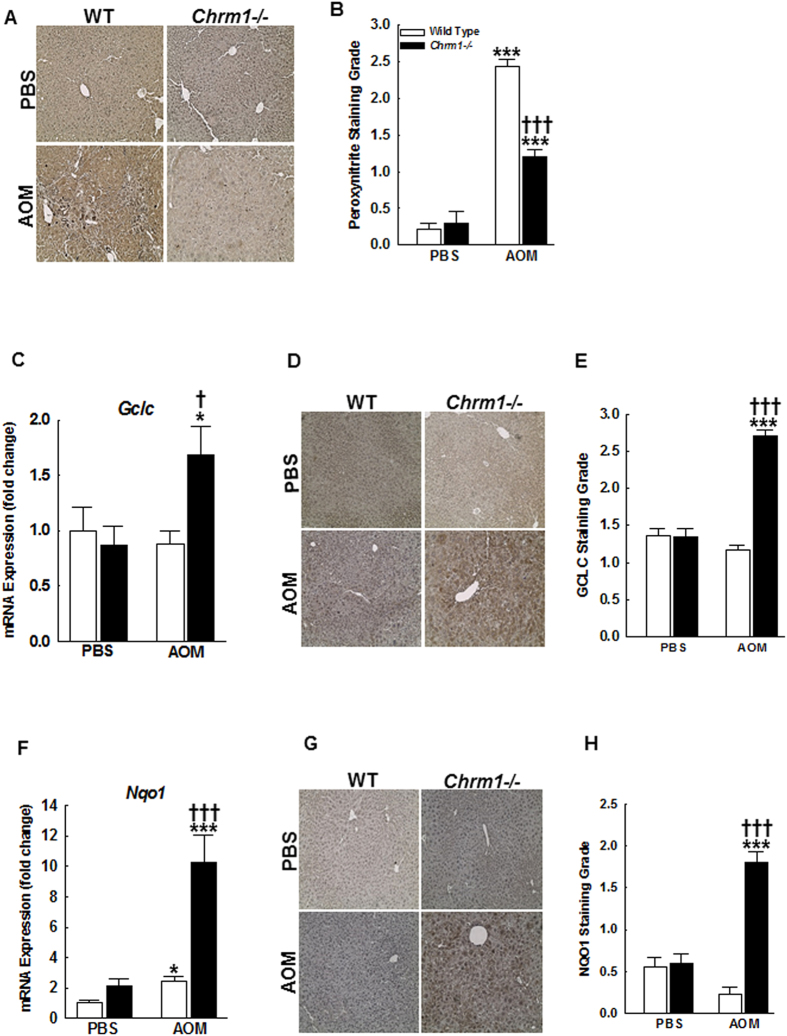
M1R deficiency enhances induction of the anti-oxidant response. (**A**) Representative 3-Nitrotyrosine-stained liver sections. (**B**) Summary data. Peroxynitrite adduct formation was reduced in the livers of AOM-treated M1R-deficient mice when compared to those from AOM-treated WT mice. (**C**) Baseline hepatic expression of *Gclc* mRNA was similar among PBS-treated WT and M1R-deficient mice. After AOM treatment, *Gclc* expression was increased in livers from M1R-deficient mice and unchanged in livers from WT mice. (**D**) Representative Gclc-stained liver sections. (**E**) Summary data. Livers from AOM-treated M1R-deficient mice had increased hepatocyte Gclc staining, which mirrored mRNA expression. (**F**) Hepatic expression of *Nqo1* appears greater in PBS-treated M1R-deficient mice compared to PBS-treated WT mice. After AOM treatment, *Nqo1* mRNA expression was increased in all mice. Compared to AOM-treated WT mice, *Nqo1* expression was markedly increased in AOM-treated M1R-deficient mice. (**G**) Representative Nqo1-stained liver sections. (**H**) Summary data. Livers from AOM-treated M1R-deficient mice had increased hepatocyte Nqo1 staining. *p < 0.05, ***p < 0.001 within genotype, and **†***p* < 0.05, **†††***p* < 0.001 within treatment group.

Previously we showed that after acetaminophen overdose, M1R-deficient mice mount a robust anti-oxidant response with up-regulation of cytoprotective genes, *Gclc* and *Nqo1*, reduction of peroxynitrite generation and reduced necrotic liver injury^14^. Oxidative stress is a promoter of hepatocyte apoptosis and driver for persistent stellate cell activation and hepatic fibrosis^37^. Hence, we hypothesized that in response to AOM, M1R-deficient mice would mount an anti-oxidant response. To test our hypothesis, we first assessed peroxynitrite adduct formation. As shown by IHC (Fig. 5A,B), AOM treatment increased hepatic peroxynitrite adduct generation in both WT and M1R-deficient mice, however, compared to AOM-treated WT mice, peroxynitrite adduct generation was reduced in the livers of AOM-treated M1R-deficient mice. Next, we assessed hepatic *Gclc* and *Nqo1* expression. *Gclc* expression was similar in mice treated with PBS; however, compared to AOM-treated WT mice, *Gclc* expression increased ~ 2-fold in AOM-treated M1R-deficient mice (*p* < *0.05*, Fig. 5C). Mirroring PCR findings, GCLC staining was increased in liver sections from AOM-treated M1R-deficient mice (Fig. 5D,E). Expression of *Nqo1* was also similar in PBS-treated mice; however, compared to AOM-treated WT mice, *Nqo1* expression increased significantly in AOM-treated M1R-deficient mice (2.42 ± 0.31 vs. 10.29 ± 1.74 fold respectively, *p* < *0.001*; Fig. 5F). IHC confirmed the PCR findings and revealed increased NQO1 staining in hepatocytes of AOM-treated M1R-deficient mice compared to those from AOM-treated WT mice (Fig. 5G,H).

### M1R deficiency reduces the survival of activated HSC

**Figure 6:**
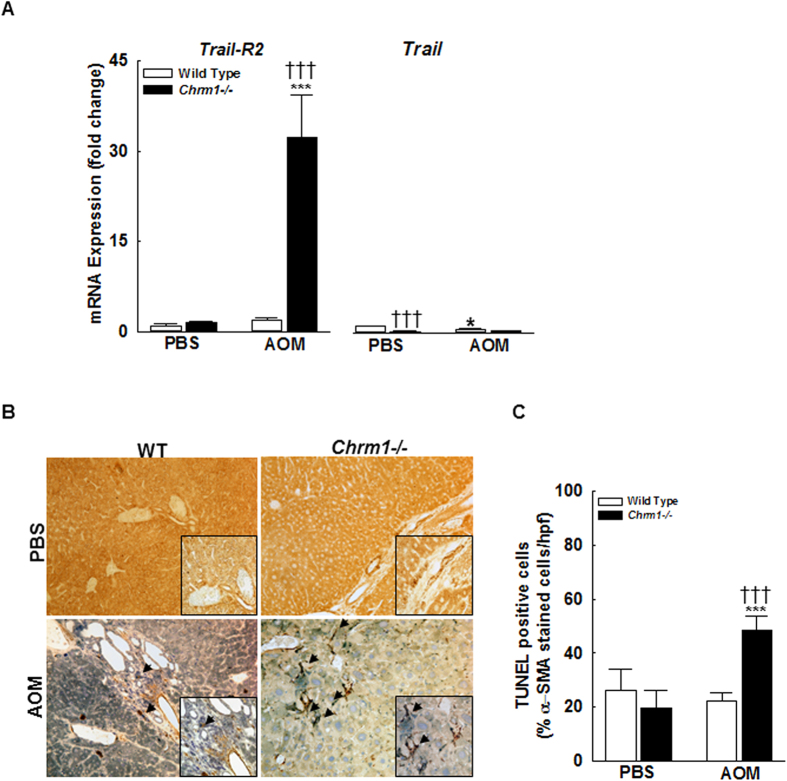
M1R deficiency augments *Trail-R2* expression in HSC and promotes HSC apoptosis. (**A**) Compared to WT mice, livers from AOM-treated M1R-deficient mice had 32-fold increased *Trail-R2* expression. In PBS-treated mice, M1R deficiency reduced *Trail* expression. After AOM treatment, *Trail* expression in WT and M1R-deficient mice was similar, although AOM exposure decreased *Trail* expression only in WT mice. (**B**) Representative photomicrographs of liver sections stained for both TUNEL and α-SMA at 200x magnification with 400x insert. Arrows indicate TUNEL (+) activated HSC. (**C**) After AOM exposure, the proportion of TUNEL-positive HSC was significantly increased in livers from M1R-deficient mice compared to those from WT mice. *p < 0.05, ***p < 0.001 within genotype, and **†***p* < 0.05, **†††***p* < 0.001 within treatment group.

As activated HSCs were reduced in M1R-deficient mice after AOM-induced liver injury, we explored the role of M1R on HSC survival. HSC apoptosis is mediated through TNF-related apoptosis inducing ligand (TRAIL)-related pathways which are not implicated in hepatocyte apoptosis^38^. In particular, activated HSC preferentially express the death receptor TRAIL-R2^39^, resulting in increased sensitivity to TRAIL-induced apoptosis. To determine the effect of M1R deficiency on TRAIL-mediated HSC death, we measured expression levels of *Trail-R2* and *Trail*. In PBS-treated mice, M1R deficiency did not alter *Trail-R2* expression. However, compared to WT mice, there was a 32-fold increase in *Trail-R2* expression in AOM-treated M1R-deficient mice (*p* < *0.001*, Fig. 6A). Compared to PBS-treated WT mice, PBS-treated M1R-deficient mice had decreased expression of *Trail* (1.0 ± 0.15 vs. 0.2 ± 0.02 fold respectively, *p* < *0.001*; Fig. 6A). After AOM treatment, *Trail* expression in WT and M1R-deficient was similar (0.53 ± 0.14 vs. 0.27 ± 0.07 fold respectively); AOM exposure decreased *Trail* expression only in WT mice (Fig. 6A).

To quantify HSC apoptosis, we stained liver sections for both α-SMA and TUNEL. With AOM treatment, the proportion of TUNEL-positive HSC was significantly increased in M1R-deficient compared to WT mice (Fig. 6B,C), revealing increased HSC apoptosis in AOM-treated M1R-deficient mice. These findings indicate that *in vivo,* M1R deficiency up-regulates *Trail-R2* expression and enhances HSC apoptosis, thus decreasing AOM-induced hepatic fibrosis.

### M1R inhibition attenuates oxidative stress-mediated hepatocyte apoptosis

**Figure 7:**
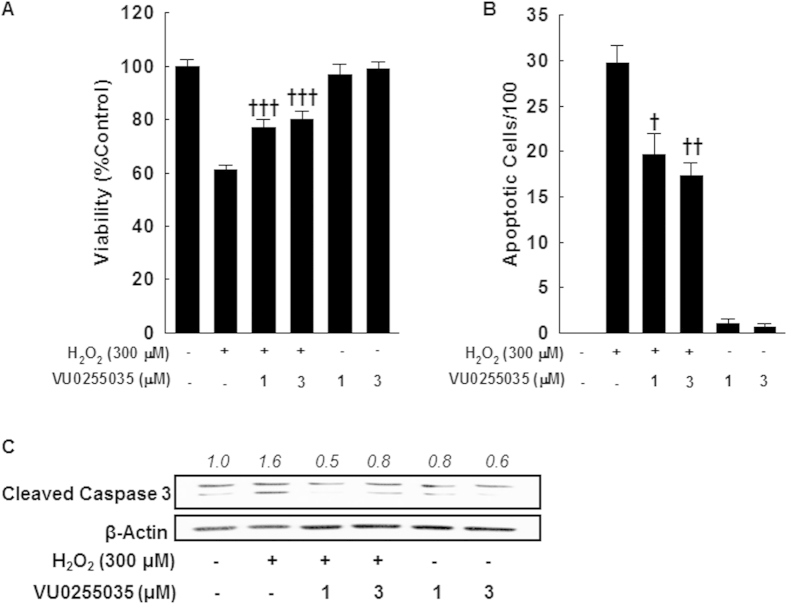
Pharmacological inhibition of M1R activity reduces oxidative stress-induced apoptosis of AML-12 hepatocytes. (**A**) Effects on AML12 hepatocyte survival were assessed using the MTT assay. Incubation with 300 μM H_2_O_2_ for 24 h reduced AML12 cell survival while co-treatment with VU0255035 (1 and 3 μM) significantly enhanced cell survival. Treatment with VU0255035 alone had no effect. (**B**) Effects on AML12 hepatocyte apoptosis were assessed by DAPI staining. Incubation with 300 μM H_2_O_2_ for 24 h increased AML12 cell apoptosis which was reduced by co-treatment with VU0255035 (1 and 3 μM). Treatment with VU0255035 alone had no effect. (**C**) Representative blots indicate that treatment of AML12 cells with 300 μM H_2_O_2_ for 24 h increased caspase-3 cleavage, which was reduced when cells were co-treated with VU0255035. All samples contained DMSO, the vehicle for VU0255035. Results are mean ± S.E.M. **†***p* < 0.05, **††***p* < 0.01, **†††***p* < 0.001 compared cells treated with only H_2_O_2_. Full-length blots are presented in Supplementary Figure 1.

Our *in vivo* data indicated that genetic ablation of M1R reduces AOM-induced oxidative stress and hepatocyte apoptosis. In the liver, M1R is predominantly expressed in hepatocytes^14^. Since hepatocytes apoptosis is a key instigator of fibrogenesis^1^, we investigated if inhibiting M1R activity could attenuate hepatocyte apoptosis. We assessed the effects of VU0255035, a novel M1R-selective antagonist with >75-fold selectivity for M1R over other muscarinic receptor subtypes, on oxidative stress-induced apoptosis in AML12 hepatocytes^40^. M1R is expressed at similar levels in AML12 cells, primary mouse hepatocytes and whole mouse liver^14^. Low-dose H_2_O_2_ (<1 mM) induces hepatocyte apoptosis^41^,^42^, whereas higher doses (>1 mM) induce necrosis. Using AML12 hepatocytes, we assessed the effect of VU0255035 on H_2_O_2_ (300 μM)-mediated cell death and cleavage of caspase-3; 300 μM H_2_O_2_ consistently induced maximal PARP cleavage (not shown). M1R inhibition in AML12 hepatocytes enhanced cell survival (Fig. 7A) and reduced cell death (Fig. 7B); this was associated with reduced caspase-3 activation (Fig. 7C). These findings are in accordance with our *in vivo* data that indicate M1R deficiency reduces AOM-induced caspase-3 activation in hepatocytes.

## Discussion

Previously, we showed that AOM treatment induces chronic liver injury in mice characterized by liver nodularity, fibrosis, ductular hyperplasia, hepatocyte apoptosis, and compensatory hepatocyte proliferation^10^. Our findings in the present study are consistent with these observations^10^,^27^. We also showed previously that M3R activity modulates the response to toxin-induced hepatic damage; M3R deficiency greatly enhanced gross and microscopic liver injury^10^. While non-selective muscarinic antagonism with scopolamine butylbromide also increased AOM-induced hepatic damage, this was much less severe than in M3R-deficient mice^10^. Together, these observations suggested to us that another muscarinic receptor subtype(s) might play a divergent role in regulating chronic liver injury. M1R are expressed in the murine and human liver, predominantly in hepatocytes, and M1R deficiency reduces *acute* liver injury in mice^14^. M1R deficiency and inhibition were cytoprotective and enhanced anti-oxidant responses in hepatocytes^14^. This led us to hypothesize that M1R might play a role in regulating chronic liver injury.

To test this hypothesis, we investigated the effects of repeated AOM treatments in M1R-deficient compared to WT mice. In short, our findings substantiated our hypothesis, AOM-treated M1R-deficient mice had markedly reduced AOM-induced liver surface nodularity and fibrosis compared to AOM-treated WT mice. Indeed, all of the major drivers of hepatic fibrosis—hepatocyte apoptosis, ductular reaction, and oxidative stress—were markedly reduced in M1R-deficient mice. M1R deficiency resulted in anti-fibrotic effects on hepatic stellate cell activation, function and survival. AOM-treated M1R-deficient mice had nearly three-fold decreased HSC activation as measured by IHC for α-SMA. While expression of PDGF and TGFβ was not altered, M1R deficiency reduced HSC activation and proliferation as a consequence of down-regulation of PDGF and TGFβ receptors. Consistent with these observations, AOM-induced α1 collagen(I) mRNA expression was reduced in M1R-deficient mice. Interestingly, after AOM exposure M1R deficiency was associated with more than 30-fold up-regulation of *Trail R2* and enhanced HSC apoptosis as indicated by α-SMA and TUNEL co-staining. These findings are consistent with previous reports in BDL and CCl_4_ murine models of liver injury in which reduced fibrosis was accompanied by HSC apoptosis^17^,^18^ – activated HSC utilize Trail R2-mediated signaling pathways to initiate apoptosis^38^,^39^.

Hepatocyte apoptosis is the fundamental event that induces stellate cell activation. We showed previously in mice that repeated AOM-treatment induces hepatocyte apoptosis. Here we show that M1R deficiency reduces AOM-induced hepatocyte apoptosis, associated with *Tnf-α* down-regulation. TNF-α is a pleiotropic ligand that induces apoptosis by activating caspases and also primes hepatocytes for proliferation. Based on the expression profiles of *Tnf-α* and cleaved caspase-3, we believe diminished hepatocyte proliferation in M1R-deficient mice is a consequence of reduced injury. Our *in vitro* experiments confirmed that inhibiting M1R in hepatocytes subjected to oxidative stress reduces caspase-3 activation and enhances cell survival.

Ductular hyperplasia is a characteristic feature of most chronic liver diseases^31^,^43^. Proliferating ductular cells express TGF-β, which induces paracrine activation of stellate cells^44^. Therefore, ductular reaction is thought to be a major propagator of fibrogenesis in chronic liver disease. TWEAK signaling was shown to play a key role on proliferating ductular cells. A recent study shows that inhibiting TWEAK signaling attenuates ductular proliferation, and also reduces pro-fibrogenic responses and promotes liver regeneration in fibrotic livers^35^. We observed markedly low basal *Tweak* expression in M1R-deficient mice, which decreased further upon treatment with AOM; this was associated with markedly reduced bile ductular proliferation. These findings confirm that in this model ductular hyperplasia mirrors the severity of fibrosis^45^, and suggests that M1R deficiency attenuates AOM-induced ductular proliferation by interfering with TWEAK signaling.

Oxidative stress plays a multi-faceted role in chronic liver injury^46^. On one hand, oxidative stress promotes hepatocyte apoptosis while on the other it promotes HSC activation^47^. Apoptotic bodies derived from dying hepatocytes directly activate stellate cells^48^,^49^,^50^. Inhibition of oxidative stress reduces hepatocyte loss and stellate cell activation *in vitro* and *in vivo* and reduces fibrogenesis in BDL mice^51^. Thus, oxidative stress is a major driver of chronic liver injury. Our findings are in-line with these observations; compared to AOM-treated WT mice, livers from AOM-treated M1R-deficient mice had reduced peroxynitrite adduct formation and up-regulated expression of *Gclc* and *Nqo1*. GCLC promotes glutathione generation which scavenges peroxynitrite and free radicals, whereas NQO1 inhibits TGF-β-mediated activation of the fibrogenic response^52^. Previously, we demonstrated that M1R deficiency or inhibition promoted anti-oxidant responses in acetaminophen-induced liver injury. Our current findings indicate that in response to injurious stimuli, in hepatocytes M1R inhibition up-regulates expression of anti-oxidant cytoprotective genes, thereby reducing parenchymal cell loss and in turn, reducing stellate cell activation.

Our study has limitations. First, the role of cholinergic signaling in modulating stellate cell behavior remains uncertain. Previous studies showed that nicotinic acetylcholine receptor (nAChR) stimulation augments stellate cell activation^53^,^54^; while another study suggested nAChR play a lesser role. Although we observed very low M1R expression in stellate cells isolated from mouse livers^14^, M2R may be involved in stellate cell proliferation^55^. Divergent findings may also result from different experimental models. In view of our previous and current findings, we believe that enhanced stellate cell deactivation observed in AOM-treated M1R-deficient mice results from reduced oxidative stress and hepatocyte apoptosis. Nevertheless, the role of M1R and M2R in modulating hepatic stellate cell physiology warrants further investigation. Second, the role of M1R in TWEAK signaling is unknown. We observed reduced ductular reaction in AOM-treated M1R-deficient mice. TWEAK plays a key role in ductular proliferation, and infiltrating immune cells in liver, predominantly macrophages, are thought be the chief source of TWEAK^56^. We observed very low basal TWEAK expression in M1R-deficient mice that was further reduced after AOM treatment. While, we suspect reduced ductular reaction in AOM-treated M1R-deficient mice is a consequence of reduced liver injury, cross-talk between cholinergic and TWEAK signaling requires further investigation.

M1R is a GPCR, which couples predominantly to G_q/11_ to induce PLC activation that results in increased intracellular calcium^57^. Muscarinic receptors are active constitutively, but little is known about their actions in hepatocytes^58^,^59^. In view of our recent^14^ and current findings, we hypothesize that M1R inhibit oxidative-stress sensors in hepatocytes, a function that is negated upon M1R deficiency or inhibition, which helps hepatocytes mount a robust anti-oxidant response when they are subjected to oxidative stress.

In conclusion, the present study indicates that M1R deficiency modulates chronic liver injury by activating anti-oxidant responses; up-regulating *Gclc* and *Nqo1*, attenuating hepatocyte death by reducing caspase-3 activation, decreasing HSC activation and proliferation to decrease fibrogenesis, and reducing ductular reaction possibly by down-regulating Tweak expression. Overall, our findings implicate M1R as a major modulator of chronic liver injury and suggest that selective inhibition of M1R activity is a therapeutic strategy against chronic liver disease that is worthy of further investigation.

## Materials and Methods

### Animals

Animal care and studies were conducted in accordance with *The Guide and Use of Laboratory Animals* prepared by the United States National Academy of Sciences (National Institutes of Health publication number 86-23, revised 1996) and approved by the Institutional Animal Care and Use Committee at the University of Maryland School of Medicine and the Research and Development Committee at the the VA Maryland Health Care System (Baltimore, Maryland). Six-week-old *Chrm1*-deficient (*Chrm1−/−*, n = 29) male mice (genetic background: 129S6/SvEv X CF1, 50%:50%) and wild type (WT, n = 25) control mice were generated as previously described and provided by Dr. Jürgen Wess^60^. Mice were housed in a pathogen-free environment with 12:12 hours light/cycle and free access to standard mouse chow and water. Mice were acclimatized for two weeks prior to experiments.

### Experimental Design

The study design is shown in Fig. 1A. Twenty WT and 24 *Chrm1−/−* mice were treated with intraperitoneal AOM (10 μg g^−1^ once weekly for 6 weeks). For treatment controls, 5 WT and 5 *Chrm1−/−* mice received intraperitoneal PBS instead of AOM. Mice were euthanized 20 weeks after the first dose of AOM or PBS. To assess cell proliferation, two hours prior to euthanasia mice were injected with 50 mg kg^−1^ 5-bromo-2′-deoxyuridine (BrdU; Sigma-Aldrich, St. Louis, MO). At euthanasia, gross liver appearance was evaluated by two investigators masked to genotype and treatment, using a previously-described grading system^10^. Livers were then harvested and portions fixed in 4% formaldehyde and stored in RNA*later* (Life Technologies, Grand Island, NY).

### Liver Histology

Formalin-fixed, paraffin-embedded tissue blocks were sliced into 5 μm sections and mounted on glass slides. The collagen content was quantified by morphometric analysis of Sirius Red-stained sections as described previously^10^.

### Immunohistochemistry (IHC)

Liver sections were deparaffinized with xylene and rehydrated with graded alcohol washes. Heat-induced antigen retrieval was performed by microwaving specimens in citrate buffer. Sections were treated with 3% H_2_O_2_ to block endogenous peroxidase activity. This was followed by incubation with normal goat serum (Vector Laboratories, Burlingame, CA) at room temperature for 1 h to prevent nonspecific protein binding. Sections were then labeled with a primary antibody to the antigen of interest and incubated overnight at 4 °C. Next, sections were incubated with biotinylated goat-derived anti-rabbit secondary antibodies at room temperature for 1 h. The avidin-biotin reaction was performed using the VECTASTAIN Elite ABC Kit (Vector Laboratories, Burlingame, CA) according to the manufacturer’s instructions. Specimens were stained using diaminobenzidine (Sigma Aldrich, St. Louis, MO) and counterstained with hematoxylin (Sigma Aldrich).

To assess cell proliferation, we used a primary antibody to BrdU (BD Bioscience, San Jose, CA, 1:100 dilution). A primary antibody to cleaved-caspase 3 was used to measure hepatocyte apoptosis (Cell Signaling Technology, Boston, MA, 1:800 dilution), and a primary antibody to α-smooth muscle actin (α-SMA) was used to assess hepatic stellate cell activation (Abcam, Cambridge, MA, 1:100 dilution). For each antibody, the number of stained cells per 1000 hepatocytes was counted in 5 randomly chosen fields at 200X magnification.

To confirm quantification of reactive bile ducts on H&E, representative unstained sections from each experimental group were assessed by IHC for cytokeratin-19 (CK19, Abcam, Cambridge, MA, 1:100 dilution). Bile ducts were counted in five fields (200X magnification) randomly selected from each section.

To quantify hepatic stellate cell apoptosis, dual staining was performed using a primary antibody to α-SMA followed by terminal UDP-nick end labelling to assess DNA fragmentation (TUNEL; Trevigen, Gaithersburg, MD). Each slide was analyzed by a masked observer, and non-parenchymal dual-stained cells were counted in 10 randomly selected fields at 200X magnification.

To assess oxidative stress, liver sections were stained with antibodies for 3-nitrotyrosine (3-NT; Life Technologies, 1:200 dilution), NAD(P)H dehydrogenase quinone 1 (NQO1; GeneTex, 1:200 dilution) and glutamate—cysteine ligase catalytic subunit (GCLC; GeneTex, 1:200 dilution). Liver sections stained for 3-NT, NQO1 and GCLC were graded semi-quantitatively in at least 10 HPF (high power field) per section by masked investigators: 0-occasional, 1-minimal, 2-moderate and 3-extensive staining.

### RNA Extraction and cDNA Synthesis

Frozen liver sections stored in RNA*later* were removed from solution with sterile forceps and submerged in 2 ml TRIzol (Life Technologies, Grand Island, NY). Tissue homogenization, phase separation, and RNA precipitation were performed according to manufacturer’s instructions. RNA quantity and purity were assessed using a Thermo Scientific 2000 Nanodrop Spectrophotometer (Thermo Scientific, Wilmington, DE); all samples had A260/A280 between 1.8 and 2.0. Total RNA (2 μg) was reverse-transcribed to cDNA using the First Strand cDNA Synthase Kit (Fermentas, Hanover, MD) with random hexamer primers.

### Quantitative Real-Time Polymerase Chain Reaction (qPCR)

qPCR was performed using the Step One Plus PCR Detection System (Applied Biosystems, Grand Island, NY). The reaction mixture contained 12.5 μL Quantifast SYBR green master mix (Qiagen, Valencia, CA), 1 μL cDNA,1 μL primer (10 pmol/μl) and 9.5 μL nuclease free water. We used a two-step thermal cycling profile: Step I (cycling step): 95 °C for 5 min, 95 °C for 10 s and 60 °C for 30 s for 40 cycles; Step II (melting curve step): 60 °C for 15 s, 60 °C 1 min and 95 °C for 30 s. The ^ΔΔ^Ct method was used to determine fold-change in gene expression normalized to glyceraldehyde-3-phosphate (GAPDH) mRNA. Respective forward and reverse primer sequences for target genes are as follows:

*α1Collagen* GATGACGTGCAATGCAATGAA, CCCTCGACTCCTACATCTTCTGA; *Fas* TTCATACTCAAGGTACTAATAGCA, TTCAGGTTGGCATGGTTG; *FasL* AAGAAGGACCACAACACAA, TAATCCCATTCCAACCAGAG; *Gapdh* ACAACTTTGGCATTGTGGAA, GATGCAGGGATGATGTTCTG; *Gclc* AACACAGACCCAACCCAGAG, CCGCATCTTCTGGAAATGTT; *Mmp-13* GTTCAAGGAATTCAGTTTCTTTATGGT, GGTAATGGCATCAAGGGATAGG; *Mmp-2* CCCTCAAGAAGATGCAGAAGTTC, TCTTGGCTTCCGCATGGT; *Nqo1* CAGATCCTGGAAGGATGGAA, TCTGGTTGTCAGCTGGAATG; *Pdgf* GGTCCCATGCCATTAACCAT, CCGTCCTGGTCTTGCAAACT; *Pdgf-R* CTTTGTGCCAGATCCCACCA, TCACTCGGCACGGAATTGTC; *Tgfβ1* GCCTGAGTGGCTGTCTTTTGA, GCTGAATCGAAAGCCCTGTATT; *Tgfβ1-R* CCACTTGCGACAACCAGAAGTC, GTCGTTCTTCCTCCACACGG; *Timp-1* GCATGGACATTTATTCTCCACTGT, TCTCTAGGAGCCCCGATCTG; *Timp-2* TTCCGGGAATGACATCTATGG, GGGCCGTGTAGATAAACTCGAT; *Tnf-α* GTGGAACTGGCAGAAGAG, AATGAGAAGAGGCTGAGAC; *TnfR* GGGCACCTTTACGGCTTCC, GGTTCTCCTTACAGCCACACA; *Trail* CAGGCTGTGTCTGTGGCTGT, TGAGAAGCAAGCTAGTCCAATTTT; *Trail-R2* ACACCAGCCATTCCAACCAT, CCTGGTTTGCATCGACACAC; *Tweak* GCCCCCTTCCTAACCTACTTT, CATGTGAACAAGCTCTGGCT.

### Cell culture

AML12 cells (a non-tumorigenic mouse hepatocyte cell line) were cultured using a 1:1 mixture of Dulbecco’s modified Eagle’s medium and Ham’s F12 medium containing ITS solution (0.005 mg/ml insulin, 0.005 mg/ml transferrin, 5 ng/ml selenium) and 10% fetal bovine serum, at 37 °C with 5% CO_2_. All the experiments were performed at 70–80% cell density.

### Cell viability assay

AML12 cells were maintained in 96-well plates for 24 h in the presence of 0.3 mM hydrogen peroxide (H_2_O_2_) in combination with vehicle (DMSO) or M1R antagonist VU0255035 (Tocris, USA). At the end of treatment, 100 μl MTT (0.5 mg/ml in culture media) was added to all wells and incubated for 150 min at 37 °C. Subsequently, MTT solution was discarded, all the wells were washed with PBS for 5 min, and 150 μl DMSO added in each well to dissolve purple formazan. Plates were kept at room temperature for 30 min with constant shaking and absorbance was read at 540 nm using a VersaMax Microplate Reader (Molecular Devices).

### DAPI staining for apoptosis

AML12 cells were treated for 24 h with 0.3 mM H_2_O_2_ and M1R antagonist VU0255035 or vehicle, and VU0255035 alone. Subsequently, cells were trypsinized and centrifuged at 1000 g for 5 min. The cell pellet was washed with PBS once, re-suspended in permeabilization buffer and allowed to sit at room temperature for 10 min. Cells were then centrifuged and fixed using 2% buffered paraformaldehyde solution containing 10 μg/ml DAPI for 10 min in the dark. DAPI-stained pellets were washed with PBS and an aliquot was transferred on a microscope slide, mounted with coverslip and apoptotic cells counted using a fluorescence microscope (excitation wavelength, 350 nm). For each sample, three different fields were examined and 100 cells were counted in each field. Apoptotic cells were identified by their small, condensed nuclei.

### Immunoblotting

After treatments, AML12 cells were collected with a scraper in cell lysis buffer (containing phosphatase and protease inhibitors). Lysed cells were centrifuged at 20000 g for 20 min and total protein in the supernatant was determined using the Bradford Assay (Sigma, USA). Protein (60 μg) from each sample was electrophoresed on 12.5% sodium dodecyl sulfate–polyacrylamide gels and transferred onto PVDF membranes (Bio-Rad, USA). After blocking with 5% skimmed milk, membranes were incubated with rabbit anti-mouse cleaved caspase-3 antibody (1:1000; Cell Signaling, USA) overnight at 4 °C with gentle shaking. The following day, membranes were washed with PBST (1X) three times for 10 min each and incubated with goat anti-rabbit HRP antibody (1:5000; Cell Signaling) for 60 min with gentle shaking at room temperature. After washing with 1X PBST (three times for 10 min each), blots were developed using chemiluminescence reagent (Bio-Rad, USA) on an autoradiography film (Genesee Scientific, USA). To confirm equivalent loading, blots were stripped and re-probed with goat anti-rabbit β-actin antibody (1:5000; Cell signaling, USA). Scanned images of blots were used to quantify protein expression using NIH ImageJ software (http://rsb.info.nih.gov/ij/).

### Statistical Analysis

All data are expressed as mean ± standard error (S.E.M.). Quantitative PCR results were normalized to those for wild-type PBS-treated controls. The normality of data distributions was assessed using the Shapiro-Wilk test. Student’s unpaired *t*-test (normally-distributed data) and the Mann-Whitney U-test (nonparametric data) were used to determine statistical significance. Linear correlations were assessed using the Pearson *r*^*2*^ test. Statistical significance was defined as *p* < 0.05 and figures were labeled: *p < 0.05, **p < 0.01, ***p < 0.001 within genotype, and †*p* < 0.05, ††*p* < 0.01, †††*p* < 0.001 within treatment group.

## Additional Information

**How to cite this article**: Rachakonda, V. *et al.* M1 Muscarinic Receptor Deficiency Attenuates Azoxymethane-Induced Chronic Liver Injury in Mice. *Sci. Rep.* **5**, 14110; doi: 10.1038/srep14110 (2015).

## Supplementary Material

Supplementary Information
