# Supplementary material for: M1 Muscarinic Receptor Deficiency Attenuates Azoxymethane-Induced Chronic Liver Injury in Mice
Source: Sci Rep. 2015 Sep 16;5:14110. doi: 10.1038/srep14110 (PMC4571652; doi:10.1038/srep14110)
Supplement: Supplementary Information [file srep14110-s1.pdf]

Title: M1 Muscarinic Receptor Deficiency Attenuates Azoxymethane-Induced Chronic Liver Injury in Mice

Authors: Vikrant Rachakonda[1], Ravirajsinh N. Jadeja[2], Nathalie H. Urrunaga[1], Nirish Shah[1], Daniel Ahmad[1], Kunrong Cheng[1], William S. Twaddell[3], Jean-Pierre Raufman[1], and Sandeep Khurana[2*].

Affiliations: [1]Division of Gastroenterology and Hepatology, University of Maryland School of Medicine, Baltimore, Maryland, 21201; [2]Section of Gastroenterology and Hepatology, Georgia Regents University, Augusta, GA 30912; [3]Department of Pathology, University of Maryland School of Medicine, Baltimore, Maryland, 21201.

Correspondence: Sandeep Khurana, M.B.B.S.

Medical College of Georgia,

Georgia Regents University,

Digestive Health Center,

Section of Gastroenterology and Hepatology,

1120 15th Street, AD2226, Augusta, GA 30912

Ph.: 706-446-4887, Fax: 706-721-0331

E-mail: skhurana@gru.edu

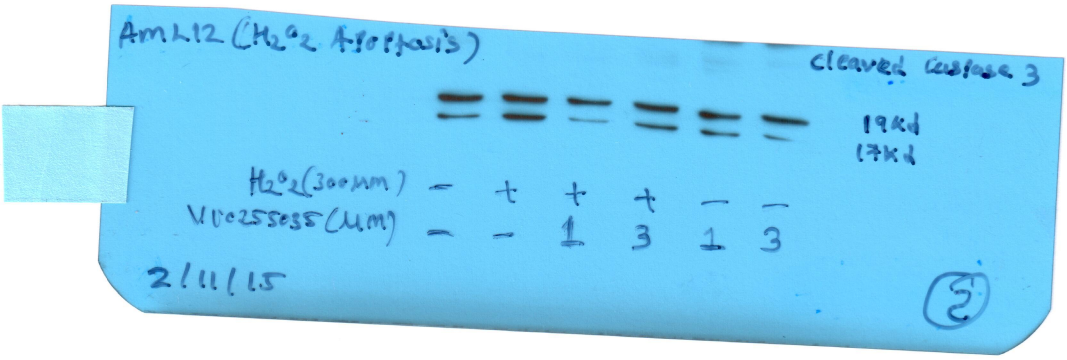
AmL12 (H2O2 Apoptosis)
cleaved caspase 3
19kd
17kd
H2O2(300uM) - + + + - -
VU0255035(uM) - - 1 3 1 3
2/11/15
2

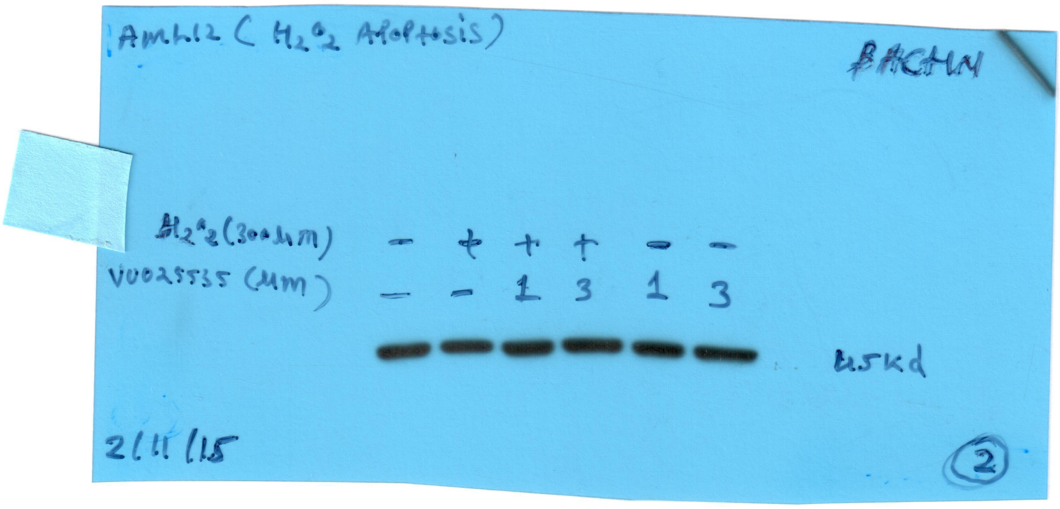
AmL12 (H2O2 Apoptosis)
H2O2(300uM) - + + + - -
VU025535 (uM) - - 1 3 1 3
2/11/15
2
